# The potential of genome editing to create novel alleles of resistance genes in rice

**DOI:** 10.3389/fgeed.2024.1415244

**Published:** 2024-06-11

**Authors:** Pankaj Kumar Singh, Basavantraya N. Devanna, Himanshu Dubey, Prabhakar Singh, Gaurav Joshi, Roshan Kumar

**Affiliations:** ^1^ Department of Biotechnology, University Centre for Research & Development, Chandigarh University, Mohali, Punjab, India; ^2^ National Rice Research Institute (ICAR), Cuttack, India; ^3^ Seri-Biotech Research Laboratory, Central Silk Board, Bangalore, India; ^4^ Botany Department, Banaras Hindu University, Varanasi, India; ^5^ Department of Pharmaceutical Sciences, Hemvati Nandan Bahuguna Garhwal (A Central University), Tehri Garhwal, Uttarakhand, India; ^6^ Department of Microbiology, Central University of Punjab, Bathinda, Punjab, India

**Keywords:** rice, resistance, CRISPR/Cas, genome editing technology, novel allele, *de novo* domestication

## Abstract

Rice, a staple food for a significant portion of the global population, faces persistent threats from various pathogens and pests, necessitating the development of resilient crop varieties. Deployment of resistance genes in rice is the best practice to manage diseases and reduce environmental damage by reducing the application of agro-chemicals. Genome editing technologies, such as CRISPR-Cas, have revolutionized the field of molecular biology, offering precise and efficient tools for targeted modifications within the rice genome. This study delves into the application of these tools to engineer novel alleles of resistance genes in rice, aiming to enhance the plant’s innate ability to combat evolving threats. By harnessing the power of genome editing, researchers can introduce tailored genetic modifications that bolster the plant’s defense mechanisms without compromising its essential characteristics. In this study, we synthesize recent advancements in genome editing methodologies applicable to rice and discuss the ethical considerations and regulatory frameworks surrounding the creation of genetically modified crops. Additionally, it explores potential challenges and future prospects for deploying edited rice varieties in agricultural landscapes. In summary, this study highlights the promise of genome editing in reshaping the genetic landscape of rice to confront emerging challenges, contributing to global food security and sustainable agriculture practices.

## 1 Introduction

Rice plays a crucial role as a major cereal crop, meeting over 23% of the world’s calorie requirements and serving as a dietary staple for half of the global population in Asia, where it occupies around 92% of the total cultivated land (Sharma et al., 2012). With the projected global population surpassing nine billion by 2050, there is a pressing need to augment overall grain production by up to 50% to meet escalating food and calorie demands (Ray et al., 2013; Prosekov and Ivanova, 2018). Achieving this target necessitates a reduction in crop losses caused by both biotic and abiotic stresses. Rice cultivation faces the challenge of approximately 70 pathogens, particularly viruses, bacteria, and fungi, resulting in substantial damage and yield reduction. Diverse major biotic pressures, including fungal sheath blight (*Rhizoctonia solani*), blast (*Magnaporthe oryzae*), false smut (*Ustilaginoidea virens*), bakanae disease (*Fusarium fujikuroi*), bacterial leaf streak (*Xanthomonas oryzae* pv. *oryzicola* or *Xoc*), bacterial blight (*X. oryzae* pv. *oryzae* or *Xoo*), and virus diseases, limit stable rice production. The cumulative yield loss from these diseases averages more than 30% (Liu et al., 2021). Consequently, the adoption of effective measures to control these diseases is vital for ensuring global food security, with the potential to significantly boost total rice production by minimizing associated losses.

Implementing the genes linked with the disease resistance characteristics in rice is the optimal strategy for disease management, while simultaneously mitigating environmental harm by reducing reliance on agro-chemicals. The development of disease-resistant rice varieties has been accomplished through diverse plant breeding approaches, encompassing conventional methods like mutagenesis, introduction of foreign genetic lines, backcross breeding with parent lines, and contemporary biotechnological techniques such as molecular marker-assisted backcross selection breeding approaches, gene stacking or pyramiding methods, etc. (Singh et al., 2018a). While these traditional breeding methods have proven highly effective in delivering superior crop varieties with elevated yields and improved characteristics, they remain the cornerstone of plant breeding. Recent advancements have facilitated the acceleration of classical breeding approaches through increased selection efficiency using selection technologies based on the marker-assisted (Collard and Mackill, 2008) and the genomic information (Desta and Ortiz, 2014). However, as our understanding of the genomic factors influencing yield and disease resistance deepens, the limitations associated with conventional breeding methods turn out to be more evident. The time-consuming and labor-intensive nature of developing resistance through conventional breeding, compounded by the challenges of linkage drag and unpredictable outcomes, hinders the ability to meet the increasing demand for quality food in the face of global hunger and malnutrition challenges (Budak et al., 2015). Additionally, the reliance on naturally occurring or randomly induced variation, limited by genetic bottlenecks during rice domestication, further impedes the classical breeding process (Shi and Lai, 2015). In contrast, genome editing technologies (GETs) offer a highly precise and controlled mutation process, allowing the immediate stacking of multiple beneficial attributes into elite backgrounds within a single generation (Zhang et al., 2014). Unlike conventional methods, direct enhancement of exiting superior varieties through genome editing avoids introducing potentially deleterious alleles through crossing and recombination. Specific artificial mutations can be introduced into the rice genome or resistance mechanism genes using GETs to generate more variation. Given the hypervariable nature of pathogens in field conditions, the continuous development of new resistant varieties remains crucial for safeguarding crops against diseases.

Advanced GETs address the constraints of traditional mutational breeding and possess the capability to swiftly impart a desired trait to any plant species and, therefore, possess significant potential to accelerate the breeding programs. Yet, comprehensive data regarding gene sequences, structures, gene functionalities, novel genes, and complex traits with quantitative trait loci (QTL) associated with desired traits are essential for the effective implementation of GETs (Jiang et al., 2012). GETs alter a particular gene with the desired trait through targeted DNA cleavage by nucleases, thereby expediting the breeding processes.

Zinc finger nucleases and transcription activator-like effector nucleases (SSEs) have emerged over the past decade as prominent gene editing tools (Kim et al., 2009). The latest progress in GET utilizing sequence-specific nucleases (SSNs) has opened up avenues for expediting the enhancement of crop traits via targeted gene editing. Within the realm of SSNs, the CRISPR-associated endonuclease Cas protein (CRISPR/Cas system) is gaining traction due to its effectiveness in generating accurate mutations. CRISPR/Cas is progressively more employed in numerous crop development programs counting rice (Miao et al., 2013; Li M. et al., 2016), wheat (Zhang et al., 2016), maize (Li J. et al., 2017), tomato (Li X. et al., 2018), soybean (Li et al., 2015), cassava (Hummel et al., 2018), citrus (Peng et al., 2017), and cotton (Zhang Z. et al., 2018).

Regarding the genetic changeability and unpredictable pathogenicity of rice pathogens, genes associated with host plant resistance have undergone co-evolution marked by significant allelic and copy number variations (Jacob et al., 2013; Singh et al., 2018b). Notably, various rice blast resistance genes are present in multiple copies, indicating a diversification driven by natural selection (Kato et al., 2007; Wang D. et al., 2014). Furthermore, even minor sequence variations among blast resistance genes, such as insufficient single nucleotide base polymorphisms (SNPs) with functional importance, can result in substantial changes in their action or resistance scale. For example, minimal amino acid variations in expected proteins of rice blast resistance genes *Pita*, *Pi2*, *Pi9*, and *Piz-t* dictate their resistance spectrum specificities (Bryan et al., 2000; Zhou et al., 2006), each bestowing broad-scale resistance counter to various rice blast strains (Wu et al., 2012). Allelic variants of a resistance (*R*) gene may exhibit differences in their cognate avirulence (*Avr*) detection specificities, providing either broad-scale or strain-specific resistances. The examination of allelic diversity in different rice blast disease resistance genes, like *Pi54* and *Piz-t*, has revealed distinct functional allelic forms with multiple sites subject to positive selection (Thakur et al., 2013a; Kumari et al., 2013; Thakur et al., 2015). Through the application of GETs, we can precisely modify existing resistance alleles or generate novel ones in rice, aiming to establish sustainable broad-spectrum resistance against pathogens.

Notably, genome editing tools have been widely employed to modify the rice genome, resulting in numerous enhanced cultivars endowed through the best selected traits like increased yield, improved quality, and enhanced stress tolerance (Zafar et al., 2020; Tabassum et al., 2021). Despite these achievements, a crucial question remains: “How much progress has been attained in rice genome editing, particularly in the realm of disease resistance, over the past decade?” While various reviews have compiled evidence of large-scale improvements through rice genome editing (Mishra et al., 2018; Mehta et al., 2020; Zafar et al., 2020; Tabassum et al., 2021), the focus on enhancing biotic stress tolerance remains relatively limited. Therefore, this article aims to systematically gather and present information on this specific aspect. By doing so, we seek to address essential questions regarding the editing of traits of interest, strategies for genomic alterations, procedures involved in nucleotide modification, the potential for achievement, and the emergence of various challenges. This systematic collation aims to enable an enhanced knowledge of the advancements and challenges in the field, providing valuable insights for future endeavors.

## 2 Resistance mechanisms in plants

The defense mechanism of plants against invading pathogens functions via two tiers of receptors. The initial layer encompasses transmembrane pattern-recognition receptors (PRRs) tasked with recognizing maintained pathogen-associated molecular patterns (PAMPs). This primary defense mechanism of plants against pathogens is referred to as pathogen-triggered immunity (PTI). An essential aspect of plant innate immunity is the initiation of PTI. The recognition of PAMPs by pattern recognition receptors (PRRs) leads to the initiation of various downstream defense-related signaling pathways. Consequently, the virulence potential of a pathogen relies on its ability to suppress PTIs through effector molecules (Speth et al., 2007). Activation of PTI initiates signaling cascades involving mitogen-activated protein (MAP) kinases, transcriptional reprogramming mediated by transcription factors like WRKY, and the production of diverse reactive oxygen species (ROS) within the host plant (Nurnberger and Kemmerling, 2009). Therefore, the initiation of signaling cascades is crucial for limiting pathogen virulence and promoting host resistance. On the contrary, effector-triggered immunity (ETI) begins with the activation by highly specialized pathogenic effectors, and their respective receptors exhibit high specificity, undergoing rigorous diversifying selection. Most of the receptors involved in ETI are part of a structurally preserved yet sequentially diverse superfamily, which comprises the nucleotide-binding site (NBS) as well as leucine-rich repeat (LRR) domains (Dangl et al., 2013). Plants typically harbor from several tens to several hundred *NBS-LRR* (*NLR*) type genes, with rice (*Oryza sativa*) boasting over 450 NLRs (Wang et al., 2019; Shang et al., 2022). The majority of NLRs are structured within tandem duplicated gene clusters, which enables the frequent emergence of new paralogs through rearrangement events among these tandem duplicates. The chromosomal double-strand breaks (DSBs), which are addressed through diverse DNA repair mechanisms such as non-homologous end-joining (NHEJ), single-strand annealing (SSA), homologous recombination (HR), and synthesis-dependent strand annealing (SDSA), lead to various outcomes such as deletions, insertions, gene conversions, and unequal or homologous recombination events (Knoll et al., 2014; Ceccaldi et al., 2016). These structural changes are regarded as significant mechanisms for creating new genes that provide resistance against diseases (Ramakrishna et al., 2002; Smith et al., 2004; Ratnaparkhe et al., 2011). More precisely, it is believed that chimeric paralogs, which arise from the recombination of divergent duplicates, constitute the principal category of new molecular factors influencing disease resistance. Precise targeted modification in the *NLRs* using GET could offer a more effective approach to enhance plant resistance against various pathogens.

The natural mutation rate appears to be adequate to confer resistance in the majority of wild populations. However, the preservation of a significant number of disease resistance genes in crops has been accompanied by a reduction in their diversity during the domestication process (Gu et al., 2015; Zheng et al., 2016), placing them at a disadvantage when confronted with rapidly mutating pathogens. Particularly, crops with widespread global cultivation face heightened vulnerability due to exposure to a diverse array of pathogens worldwide. To counter this susceptibility, the potential of genome editing emerges as a promising avenue for creating novel alleles of resistance genes in rice. This approach holds the potential to fortify biotic stress tolerance and enhance the resilience of these agriculturally significant crops on a global scale.

## 3 Overview of GETs

Genome editing, which emerged from genetic engineering in the 1970s and was propelled by the discovery of meganucleases in the late 1980s (Hsu et al., 2014; Chakraborty et al., 2017), enables precise modifications such as insertions, deletions, or substitutions of single bases or sequences (Ceasar et al., 2016; Okamoto et al., 2019; Wada et al., 2020). This technology, instrumental in gene inactivation or knockout, has rapidly advanced, offering versatile applications in both *in vitro* and *in vivo* contexts (Bortesi and Fischer, 2015). By inducing targeted alterations through double-stranded DNA breaks, repaired via various mechanisms such as homology-directed repair (HDR)/homologous recombination (HR) or non-homologous end-joining (NHEJ) depending on cell types, genome editing holds immense potential for tailored genetic modifications (Mao et al., 2008; Lieber, 2010). In NHEJ, fragmented ends reattach with nucleotide base insertions or deletions, disrupting gene function (Rouet et al., 1994). Conversely, HDR uses homologous nucleotide sequences from a donor template for precise repair with specific genomic modifications (Puchta, 2005). HDR repairs at a slower pace and with lower frequency than NHEJ, posing challenges in plant applications (Mengiste and Paszkowski, 1999; Miyaoka et al., 2016). DNA base-specific nucleases like zinc finger nucleases (ZFNs), transcription activator-like effector nucleases (TALENs), and CRISPR-associated proteins (Cas variants) are employed to create gene knockout mutants or facilitate gene replacement (Kim et al., 2009; Mishra et al., 2018; Takatsuka et al., 2022). Initially discovered in bacterial, yeast, and mammalian systems, these nucleases are now employed in various crop plants for trait improvement (Randhawa and Sengar, 2021), allowing for the creation of gene knockout mutants or the facilitation of gene replacement.

### 3.1 Zinc finger nucleases (ZFNs) technology

ZFNs are proteins that have been engineered with a zinc finger domain located at the N-terminal and an endonuclease domain situated at the C-terminal end, crucial for specific DNA sequence recognition and cleavage (Kim et al., 1996; Porteus and Carroll, 2005). This former domain enables the precise recognition of the designated DNA bases, and the *Fok*I restriction enzyme’s endonuclease domain ensures cleavage of specific DNA sequences (Bitinaite et al., 1998). Heterodimerization of FokI RE is essential for ZFN functionality, requiring two ZFNs to dimerize and bind both DNA strands. In ZFN, a consecutive series of three to six zinc fingers is arranged, with each zinc finger recognizing approximately 3 base pairs of DNA, and directs the nuclease to cut a specific genomic site (Wolfe et al., 2000). ZFNs find broad application in tailored genome engineering across diverse organisms (Urnov et al., 2010). Although initially applied for gene knockout in mice, ZFNs have limited applications in agriculture for crop improvement, mainly in crops like Arabidopsis, tobacco, and maize (Petolino, 2015). However, their off-target binding and the challenging and time-consuming nature of ZFN molecule design make them inefficient and less cost-effective for specific mutations (Pattanayak et al., 2011).

### 3.2 Transcription activator-like effector nuclease (TALEN) technology

Zinc finger nucleases (ZFN) held the position as the predominant programmable site-specific nuclease until the emergence of the DNA sequence binding effector protein, i.e., transcriptional activator-like effector (TALE), derived from *Xanthomonas*, a bacterium known for its pathogenicity in plants (Boch et al., 2009; Moscou and Bogdanove, 2009). TALE is primarily responsible for regulating disease susceptibility associated (*S*) genes in rice. It is composed of a transcriptional activation domain (AD) at the C-terminal and a nuclear localization signal signature (NLS) for transcriptional regulation, along with a crucial tandem repeats acting as a DNA sequence binding domain (DBD), and a translocation signal sequence at the N-terminal (Jankele and Svoboda, 2014). The DBD contains a short amino acid (33–35 long) repetitive stretch, with peptides at the 12th and 13th positions, termed repeat-variable di-residues (RVDs), exhibits significant variability and is accountable for the specific detection of nucleotide bases (Mak et al., 2013). Utilizing the sequence-specific binding characteristic of the DBD has resulted in the creation of a novel gene editing (GE) technique known as transcription activator-like effector nuclease (TALEN).

Like ZFN, TALENs are tailored by merging the DBD of TALE with the *Fok*I, a restriction enzyme that identifies asymmetric DNA sequences and cuts them outside of their detection sequence (Joung and Sander, 2013). However, TALEN design is simpler than ZFN, as TALE repeat sequences exhibit precision in targeting individual sites within a genome. Unlike ZFN, constructing a long array of DBD does not necessitate the multimerization of the repeat sequence, making TALEN engineering easier and less time-consuming (Gaj et al., 2013). RVDs in TALE repeat regions aid in recognizing specificity for various binding targets, allowing flexibility in designing TALENs for a broader range of potential target sites compared to ZFNs’ targeting sites. TALENs have found extensive application in genome editing across various plant species. Additionally, TALEs binding with gene activators and receptors, beyond nucleases, creates effective engineered transcriptional controllers for preferred gene expression level. In spite of TALENs’ benefits in high target specificity and little off-target effects than ZFNs exhibit, the intricate repetitive pattern within the DBD part of the TALE protein limits their application in editing multiple genomes with target specificity, and protein modification remains challenging (Chattopadhyay et al., 2022). To address these issues, the utilization of programmable RNA-directed DNA endonucleases for genome editing has become increasingly popular.

### 3.3 CRISPR/Cas technology

CRISPR, short for clustered regularly interspaced short palindromic repeats, denotes a distinct nucleotide sequence discovered within prokaryotic genomes. It structurally consists of CRISPR repeat-spacer arrays. This structure forms a locus containing conserved protein-coding genes associated with CRISPR (Cas). These genes are intricately involved in prokaryotic acquired defense mechanisms against bacteriophage invasions, akin to eukaryotic RNA interference (RNAi), thus providing genetic memory from previous encounters (Makarova et al., 2006; Bhushan et al., 2021). CRISPR utilizes small CRISPR RNA (crRNA) molecules synthesized from inherited genetic long-term memory as guide RNAs to cleave the viral genome (Barrangou et al., 2007; Brouns et al., 2008). This customizable feature of CRISPR has led to the development of the popular RNA-directed DNA endonuclease-centered genome editing technique known as the CRISPR/Cas system. CRISPR/Cas-centred genome editing relies on RNA-DNA sequence-pairing to precisely manipulate host DNA bases. It has emerged as a novel approach for genome manipulation across various species, including plants (Mushtaq et al., 2021). This technique offers robustness, simplicity, cost-effectiveness, ease of application, and high versatility compared to ZFN and TALEN (Ahmad et al., 2018). Its accuracy remains high even in multiplex genome editing, allowing for the simultaneous targeting of multiple genes (McCarty et al., 2020). Demonstrated in model plant systems (such as tobacco and arabidopsis) and diverse crops (including tomato, rice, maize, soybean, wheat, and potato), along with woody plant systems (like poplar, apple), CRISPR/Cas facilitates resilient trait improvements, addressing challenges related to both quantity and quality enhancement, and alleviating abiotic and biotic stresses (Arora and Narula, 2017; Jaganathan et al., 2018; Montecillo et al., 2020). The CRISPR/Cas technology induces dsDNA breaks at specific genome sites using a dual guide RNA system crafted to match target sequences. Watson–Crick base pairing enables binding with one strand of genomic DNA, facilitating cleavage of dsDNA by Cas9 endonucleases (Jiang and Doudna, 2017). Cellular repair mechanisms, comprising NHEJ or HDR, then mend the double-stranded breaks, resulting in genomic modifications like mutations through deletion and insertion (Shin et al., 2017). The application of these technologies for enhancing resistance to biotic stress in plants represents a promising avenue for improving rice crops.

## 4 Genome editing approaches for disease resistance

**FIGURE 1:**
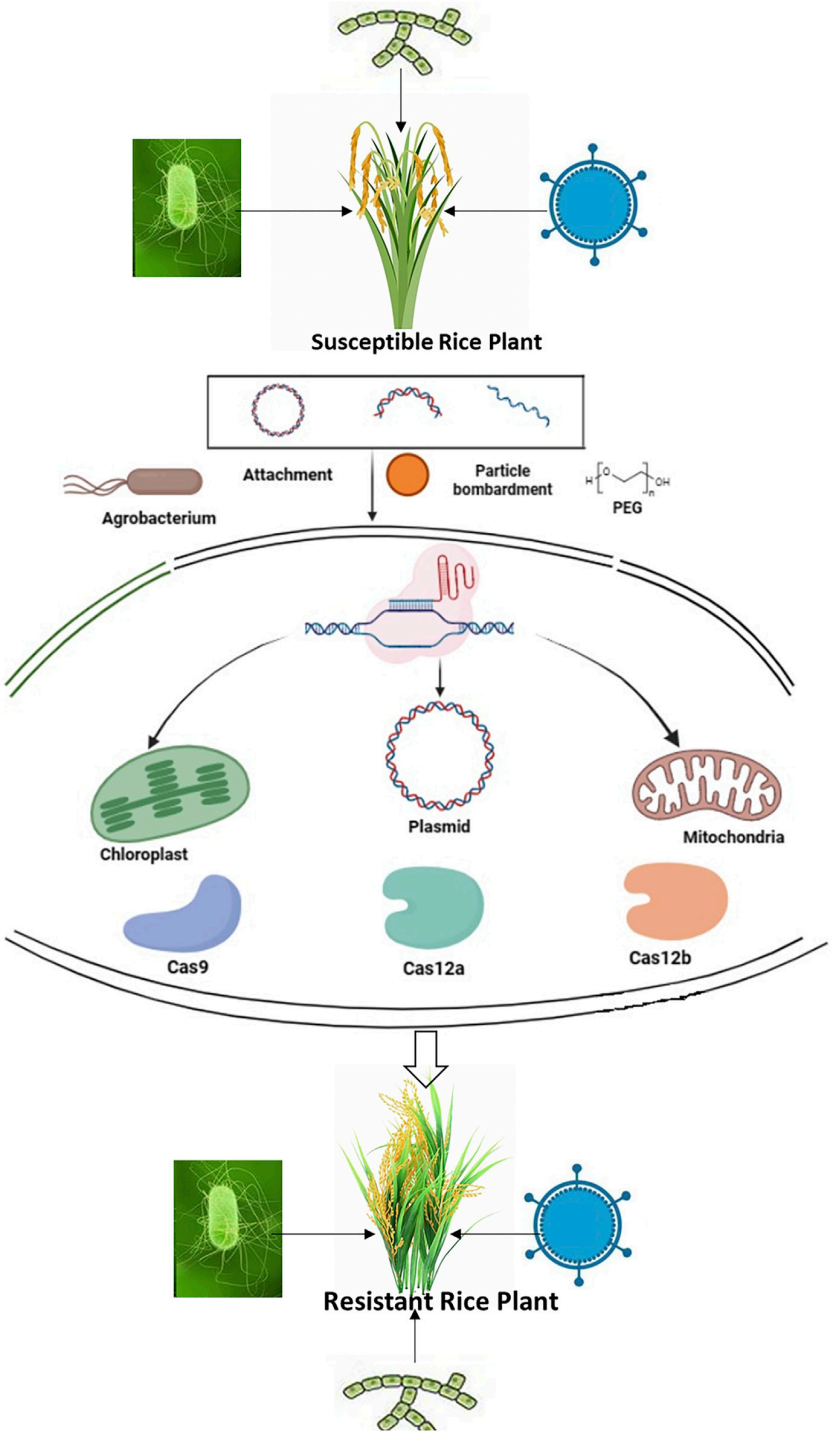
The fundamental process of employing CRISPR/Cas technology for the modification of specific genes.

The interaction between plants and pathogens is a dynamic process that involves various stages such as attachment, recognition, penetration, pathogen proliferation, and disease development. At each step, molecular mechanisms play a crucial role in regulating gene function, ultimately determining whether the outcome will be resistance or susceptibility (Singh et al., 2018a). Understanding these mechanisms is essential for gaining insights into the evolution of both hosts and pathogens, which can inform strategic pathogen control. In the age of genome editing with systems biology, exploring these technologies becomes imperative for optimizing plant-pathogen interactions with the goal of achieving sustainable resistance (Chattopadhyay et al., 2022). The major genome editing approaches can be utilized for modifying pathogen targets in crops, controlling the host immune response, and bolstering plant immunity by intervening in pathogen-plant interactions, as demonstrated in Figure 1 (Schenke and Cai, 2020).

### 4.1 Altering pathogen targets in plants

Modifying pathogen targets involves functional knockout of host susceptibility (*S*) genes, as demonstrated in rice, cucurbits, and tomato with *eIF4E* and *eIF(iso)4E* knockout for improved immunity to plant viruses (Chandrasekaran et al., 2016; Macovei et al., 2018; Moury et al., 2020; Yoon et al., 2020). However, complete disruption of pleiotropic genes may adversely affect plant health and harvest. Alternatively, crafting cis-regulatory elements in *S* gene promoters offers broad-spectrum defense that is derived by the mechanism of effector binding elements (EBEs) removal from the *S* genes in the rice crop (Oliva et al., 2019). Modification of *S* gene coding sequences through precise genome or base editing, using CRISPR/Cas9 and base editors, introduces single-base changes to create single nucleotide polymorphisms in pathogen effector binding sites, thus reducing fitness costs. Additionally, altering amino acids in surface receptor proteins, such as AtBAK1 and RIN4, can prevent evasion of secreted pathogen effectors without completely abolishing their natural function (Rodriguez et al., 2016; Li et al., 2019).

### 4.2 Regulating host plant immune response

Regulating the host immune response entails knocking out negative regulators’ transcription factors (TFs), as illustrated by the deletion of *OsERF922* in rice, which reduces abscisic acid accumulation and enhances resistance to blast pathogens (Wang et al., 2016). Similarly, modifying central regulators like *NPR1*, *NPR3*, etc., can lower infection levels; for instance, CRISPR/Cas-directed alteration in *OsNPR3* enhances the resistance mechanism in cocoa against *Phytophthora tropicalis* (Fister et al., 2018). Utilizing genome editing approaches to reduce the expression of non-coding RNAs (siRNAs and miRNAs) has been demonstrated, such as CRISPR/Cas9 editing of *OsMIR408* and *OsMIR528* in rice (Zhou et al., 2017). Alternatively, modifying plant defense through metabolic pathway engineering using genome editing approaches has been confirmed in plants like tomato and *Opium poppy* (Alagoz et al., 2016; Li R. et al., 2018). Genome editing tools are effectively employed for in planta intervention, managing the growth of pathogen infection in the plants. Primarily validated against DNA and RNA viruses (Beet curly top virus, Tomato yellow leaf curl virus, Cucumber mosaic virus) through CRISPR/Cas protein expression (Cao et al., 2020; Varanda et al., 2021), this strategy has now been extended to disrupt bacterial genomes using CRISPR nucleases, confirming a new resistance process in plants (Lee et al., 2021).

### 4.3 Enhancing plant immunity by understanding mechanism of host-pathogen interaction

Lastly, it should also be feasible to alter specific amino acids within a plant’s focused protein that are crucial for detection and cutting by pathogen effector molecules. For instance, effector proteases from a bacterium *Pseudomonas syringae*, such as AvrPphB chopping the signaling constituents PBL- 1, 2, 6, PBS1, and BIK1, or HopB1 slicing the co-receptor BAK1, could be targeted (Zhang et al., 2010; Li L. et al., 2016). A mere change of one amino acid within the cleavage motif disrupts the cleavage functionality mediated by AvrPphB. Nonetheless, a dual alteration in BIK1 protease at the positions G230A and D231A revealed a central-negative effect on flg22-triggered defense related signaling mechanism, suggesting the functional significance of the altered residues in the protease (Zhang et al., 2010). Hence, it is crucial that the change in amino acid does not entirely eliminate the inherent working mechanism of the targeted protein. Conversely, it is also plausible to increase the disease reaction through nucleotide alterations, which is reported for a resistance gene in wheat plant (Stirnweis et al., 2014). Dual amino acids substitution within the NBS domain increased the defense induced hypersensitive response (HR), thus enhancing protection towards powdery mildew disease. RIN4 serves as a focused molecule for four bacterial effectors, at minimum, two of them trigger hyperphosphorylation of RIN4, reducing the RIN4-ROC1 binding interface (Rodriguez et al., 2016). Some hyperphosphorylated amino acids could be interchanged to attenuate effector-elicited vulnerability. For instance, the 141th serine phosphorylation of RIN4 triggered by FLS2 activation is vital for the immune suppression, while the phosphorylation of threonine at position 166 is induced by another effector molecule AvrB (Chung et al., 2014). Moreover, the AvrB also inhibited pathogen-triggered callose deposition in wild type plants, whereas this immune suppression was not observed in mutant plants expressing RIN4 (T166A), which cannot be phosphorylated. This illustrates that numerous factors must be considered, but generally, substituting amino acid residues in the effector targets should make plants more immune. In this regard, precise nucleotide excision by ABEs or CBEs will emerge as a powerful technique, allowing researchers to swap particular amino acid residues, thereby disrupting interactions between effectors and their targets.

## 5 Enhancing disease resistance in rice

Progress in genome editing tools has expanded the scope of enhancing rice investigation and development, providing scientists with innovative pathways to cultivate new elite varieties that not only boast higher productivity but also prioritize environmental sustainability. The rice genome’s compact size, coupled with its efficient transformation techniques, abundant genetic reservoirs, and genomic resemblance to other cereals, positions it as an exemplary model system for conducting functional genomics inquiries. In the recent past, rice has emerged as a pivotal platform for evaluating the effectiveness of diverse genome editing methodologies and delving into gene functions to fortify rice enhancement efforts (Li et al., 2012; Feng et al., 2018; Zafar et al., 2020).

One such powerful approach for enhancing disease resistance in plants is knocking out genes using CRISPR technology. By targeting and disabling specific genes associated with susceptibility to pathogens, CRISPR can create plants that are more resistant to diseases. For instance, knocking out a susceptibility gene (*OsERF922*) in rice has been shown to significantly improve resistance to blast disease (Zhou et al., 2022). This method enables precise and efficient development of disease-resistant plant varieties, contributing to more sustainable agricultural practices. Furthermore, CRISPR technology can modify promoter regions to upregulate or downregulate target gene functions, thereby achieving disease resistance and desirable phenotypes in plants (Zhang H. et al., 2018). Enhancing complex plant traits often requires combining precise base editing with gene knockout technologies to simultaneously modify multiple genes (Zhu and Zhu, 2022). The following section discusses recent trends in disease resistance achieved using the CRISPR/Cas system against various viral, bacterial, and fungal pathogens.

### 5.1 Rice resistance against viral diseases

Rice yield suffers significant losses due to various viruses, with rice tungro disease posing a particularly severe threat in Southeast Asia (Bunawan et al., 2014). Managing this disease through conventional methods is challenging due to simultaneous infections by two distinct viruses, tungro bacilliform, and tungro spherical. The scarcity of suitable resistance sources hampers the utilization of resistant genotypes, making genetic engineering an attractive alternative. Extensive research highlights the importance of certain host factors, such as *eIF4G* and *eIF(iso)4G*, in the progression of plant diseases caused by viruses. RNA viruses exploit such host factors during infection for protein translation from transcript and to control host replication mechanism for their multiplication (Hwang et al., 2013). Consequently, these host factors have been targeted and mutated in various plants to obtain resistance against different RNA viruses (Chandrasekaran et al., 2016; Pyott et al., 2016). Similarly, *eIF4G* also assists tungro spherical virus during infection, and a single nucleotide polymorphism in the rice host factor *eIF4G* (in 9th exon), affecting the conserved amino acids Y1059V, 1060V 1061, contributes to the resistant characteristics (Lee et al., 2010). Moreover, mutations in the NL region of the eIF4G factor are also associated with viral resistance in rice (Macovei et al., 2018). Similarly, another mutation in the coding region of the *eIF4G* confers resistance in rice against a different virus (black-streaked dwarf virus) using CRISPR/Cas9 system (Table 1). However, this alteration does not confer resistance in rice against stripe virus (Wang et al., 2021). The black-streaked dwarf virus is a rice-infecting plant RNA virus. Plant RNA viruses display significant diversity, with their genetic compositions evolving rapidly. Consequently, it is imperative to engineer a CRISPR/Cas system capable of conferring robust and broad-spectrum immunity against these RNA viruses. The effective targeting of viral RNA genomes in model plants has paved the way for the application of this potent technology in enhancing viral resistance in crop plants (Aman et al., 2018a; Aman et al., 2018b). Zhan et al. (2019) achieved noteworthy success in using the CRISPR/Cas13 system to effectively hinder the replication of potato virus Y (PVY) in potato plants. Likewise, Zhang T. et al. (2019) employed CRISPR/Cas13 to develop transgenic rice varieties resistant to southern rice black-streaked dwarf virus and rice stripe mosaic virus. Genome editing thus emerges as the most suitable tool for rapidly generating plant virus-resistant genotypes.

**TABLE 1:** Overview of genes edited for the disease resistance in rice.

Gene	Disease resistance	Genome editing tool	References
*OsSWEET14* (*Os11N3*)	Resistance against *Xanthomonas oryzae*	TALENs	Li et al. (2012)
*OsSWEET14*	Resistance against *X. oryzae*	TALENs	Blanvillain-Baufume et al. (2017)
*Os09g29100*	Resistance against *X. oryzae*	TALENs	Cai et al. (2017)
*OsSWEET11/14*	Resistance against *X. oryzae*	CRISPR/Cas9	Jiang et al. (2013)
*OsSWEET13*	Resistance against *X. oryzae*	CRISPR/Cas9	Zhou et al. (2015)
*OsERF922*	Resistance against *Magnaporthe oryzae*	CRISPR/Cas9	Wang et al. (2016)
*OsBsr-d1*	Resistance against *M. oryzae*	CRISPR/Cas9	Li et al. (2017b)
*OsCul3a*	Resistance against *M. oryzae* and *X. oryzae*	CRISPR/Cas9	Liu et al. (2017)
*OseIF4G*	Resistance against rice tungro spherical virus (RTSV)	CRISPR/Cas9	Macovei et al. (2018)
*OsSEC3A*	Resistance against *M. oryzae*	CRISPR/Cas9	Ma et al. (2018)
*OsPi21*	Resistance against *M. oryzae*	CRISPR/Cas9	Li et al. (2019)
*OsXa13*	Resistance against *X. oryzae*	CRISPR/Cas9	Li et al. (2019)
*Os8N3*	Resistance against *X. oryzae*	CRISPR/Cas9	Kim et al. (2019)
*OsSWEET11/14*	Resistance against *X. oryzae*	CRISPR/Cas9	Xu et al. (2019)
*OsSWEET13/14*	Resistance against *X. oryzae*	CRISPR/Cas9	Oliva et al. (2019)
*crRNA–LshCas13a*	Resistance against southern rice black-streaked draft virus (SRBSDV)	CRISPR/Cas13	Zhang et al. (2019a)
*crRNA–LshCas13a*	Resistance against rice stripe mosaic virus (RSMV)	CRISPR/Cas13	Zhang et al. (2019a)
*OsCul3a*	Resistance against *M. oryzae* and *X. oryzae*	CRISPR/Cas9	Gao et al. (2020)
*OsXa13*	Resistance against *X. oryzae*	CRISPR/Cas9	Li et al. (2020c)
*Pi21/Bsr-d1/Xa5*	Resistance against M. oryzae and X. oryzae	CRISPR/Cas9	Tao et al. (2021)
*OsDjA2*	Resistance against *M. oryzae*	CRISPR/Cas9	Tavora et al. (2022)
*OsERF104*	Resistance against *M. oryzae*	CRISPR/Cas9	Tavora et al. (2022)
*Bsr-d1/Pi21/ERF922*	Resistance against *M. oryzae* and *X. oryzae*	CRISPR/Cas9	Zhou et al. (2022)
*OsSWEET11a/11b*	Resistance against *X. oryzae*	CRISPR/Cas9	Schepler-Luu et al. (2023)
*OsHDT701*	Resistance against *M. oryzae* and *X. oryzae*	CRISPR/Cas9	Mathsyaraja et al. (2024)

### 5.2 Rice resistance against bacterial diseases

Bacterial leaf blight disease poses a formidable challenge to rice cultivation, primarily due to the extensive variability of the *X. oryzae pv oryzae* (*Xoo*) pathogen, which complicates management efforts (Chattopadhyay et al., 2017). Host resistance remains the most effective strategy to combat this challenge, with the identification of 44 resistant (*R*) genes to date (Kumar et al., 2020). Notably, approximately 35% of these *R* genes exhibit recessive alleles (Zhang and Wang, 2013; Hu et al., 2017). However, ironically, certain recessive *R* alleles like *xa- 1*, *4*, *21*, alongside dominant *R* alleles like *Xa- 5*, *13*, serve as susceptibility factors. They facilitate disease progression by supporting the *Xoo* pathogen (Hu et al., 2017). This is achieved through the pathogen’s utilization of host machinery via its TALE effectors, to stimulate the expression of susceptibility genes (*S* genes), among which the *OsSWEET* family plays a crucial role by providing sucrose/sugar to the pathogen (Streubel et al., 2013; Gupta et al., 2021). Specific recognition of effector binding element sequences within the promoter region of *OsSWEET* genes by pathogen effectors (TALEs) initiates disease onset (Oliva et al., 2019). Therefore, modifying the *OsSWEET* promoter sequences emerges as a pivotal strategy for establishing comprehensive resistance to *Xoo*. Mainly, genome editing using TALENs focused on modifying rice *S* genes. Specifically, *OsSWEET 13* and *14* were targeted (Table 1). The engineering primarily involved the promoter sequence, which contained the *AvrXa7* effector binding element (EBE) in the *OsSWEET14*. This modification led to boosted resistance against *Xoo* strains that secrete AvrXa7 (Li et al., 2012). Likewise, enhancing the disease immunity in rice variety Nipponbare or Kitaake against *Xoo* strains bearing the *Tal5(F)* or *AvrXa7* effector was achieved by editing the promoter of *OsSWEET13*, which contained EBEs for *TalC*, *Tal5*, and *AvrXa7* effectors. However, this modification did not confer resistance against *TalC* effector (Blanvillain-Baufumé et al., 2017). Subsequently, the rapid advancement in CRISPR/Cas-mediated genome editing facilitated the precise sequence editing of *S* gene promoters (*OsSWEET 11* and *14*) to boost host plant immunity (Jiang et al., 2013). Therefore, CRISPR/Cas9 technology was repeatedly employed to target cis-elements within the promoters of *S* genes, aiming to safeguard rice against a range of *Xoo* strains (Zhou et al., 2015; Kim et al., 2019; Oliva et al., 2019; Xu et al., 2019; Zafar et al., 2020; Duy et al., 2021). Additionally, TALEN and CRISPR technologies were utilized to elucidate the function of new genes such as *Xa23-Ni* and *Xa10-Ni* to *Xoo* strains (Wang et al., 2017). Similarly, bacterial leaf streak (BLS) disease, caused by another *X. oryzae* pathotype *oryzicola* (*Xoc*), poses a serious threat to the rice crop. It employs different effectors to control host *S* genes and facilitate subsequent disease development. For instance, *Tal7* is a *Xoc* effector that suppresses AvrXa7-Xa7-mediated defense by activating the *Os09g29100* gene. Engineering the effector Tal7 interacting region (EBEtal7) of the *Os09g2910*0 promoter via TALEN enhances resistance to BLS pathogens (*Xoc* strain RS105) by suppressing AvrXa7-Xa7-mediated defense (Cai et al., 2017). Editing of promoters in additional *S* genes aimed to boost resistance in rice. This was shown through the disruption of the EBETal5d/Tal2g segment within the promoter of the *OsSULTR3;6* gene. This alteration conferred increased immunity in the rice line IRBB10 against *Xoc* strains containing either the *Tal5d* or *Tal2g* effectors (Xu et al., 2019). Furthermore, efforts to attain comprehensive protection from both *Xoc* and *Xoo* pathogens via CRISPR/Cas9 GET have shown promising results (Jiang et al., 2013), with mutant lines displaying broad-spectrum resistance without compromising normal agronomic traits, including yield (Kim et al., 2019). These findings highlight the promise of cutting-edge GETs for investigating and strengthening rice resistance towards different pathogenic bacteria.

### 5.3 Rice resistance against fungal diseases

Rice faces significant challenges from various fungal pathogens, leading to considerable yield losses (Singh et al., 2016). Among these, rice blast presents a severe menace, accountable for roughly 30% of global crop losses. The hemibiotrophic characteristic of the blast causing fungus (*M. oryzae*) exacerbates its extensive and catastrophic impact, resulting in the frequent occurrence of new variant races and speedy disintegration of host resistance (*R*) genes (Ning et al., 2020). Consequently, depending entirely on the implementation of newly discovered *R* genes, which alter the pathogen population structure, becomes increasingly challenging. Understandably, there is a growing demand for alternative options. Various strategies have been employed to confer blast disease protection in rice, with gene editing emerging as the really advanced approach for increasing protection towards diverse rice fungal pathogens. Primarily, rice genes such as *OsMPK5*, encoding stress-sensitive mitogen-activated protein kinase, were aimed to establish genome editing strategy in rice using RNA-guided editing approach. Employing the CRISPR/Cas9 platform, three guide RNAs were crafted to induce mutations in *OsMPK5*, leading to comprehensive disease protection in the rice variety Nipponbare (Table 1). Mutated protoplasts exhibited a mutation frequency ranging from 3% to 8% (Xie and Yang, 2013). Subsequently, different gene editing tools were used to knockout various disease susceptibility factors to achieve broad-spectrum defense. For instance, the negative controller of defense mechanisms in rice, *OsERF922TF*, was targeted for knockout using CRISPR/Cas9 platform (Wang et al., 2016). Binary vector carriers delivering the Cas9/sgRNA cartridge induced mutations and insertions-deletions (InDels) in Kuiku131 rice plants, yielding a 42% mutation rate in the transgenic T0 generation. This targeted gene knockout reduced abscisic acid accumulation, enhancing defense mechanisms without affecting other agronomically beneficial traits, demonstrating the precision of gene editing. Employing Cas9/multi-target sgRNA vector constructs, genome editing targeted multiple base positions in the OsERF922 sequence, increasing mutation efficiency and improving defense against the blast pathogen (Wang et al., 2016). Changes made in the OsSEC3A sequence, encoding a subunit for the exocyst protein complex, using the CRISPR/Cas9 platform targeted multiple sites within the gene, resulting in upregulation of defense-related genes and increased resistance to the blast fungus. However, it also caused stunted growth and lesion-like phenotypes (Ma et al., 2018). A similar method induced mutations in the resistance Pi21 gene, enhancing rice immunity against blast disease without affecting key agronomic traits (Nawaz et al., 2020). A BSR-D1 gene responsible for susceptibility factor was also selected for mutation through CRISPR/Cas9 platform to stimulate rice immunity against blast disease. The modification of the cis-regulatory element through single base mismatch led to enhanced interaction of *MYBS1* TF, resulting in the suppression of peroxidase expression and subsequent augmentation of H_2_O_2_ production (Li W. et al., 2020). Likewise, the functional significance of a newly discovered gene, Perox3, involved in peroxidase synthesis, in blast disease resistance was investigated by knocking it out using CRISPR/Cas9 platform in rice (Zhu et al., 2020).

In addition to blast disease, rice sheath blight poses significant challenge in rice production. In order to develop a wide-ranging defense mechanism against this disease, successful mutation of the *OsPFT1* gene in variety ASD16 was achieved via CRISPR/Cas9 platform, with the functional evaluation of the mutated lines pending determination (Shah et al., 2019). Besides targeting host genes, a range of fungal pathogen genes were also addressed to elucidate the roles of gene in the pathogenicity. For example, the removal of two *USTA* and *UvSLT2* genes of the false smut fungal pathogen (*U. virens*), respectively encode Ustiloxin and MAP kinase, resulted in two significant outcomes: hindrance in Ustiloxin formation and raised sensitivity to cell wall disruption. These changes consequently led to diminished fungal pathogen virulence to rice (Liang et al., 2018). Similarly, disrupting the blast fungal scytalone dehydratase gene using CRISPR/Cas9 affected melanin synthesis and appressoria development (Yamato et al., 2019), thereby reducing rice infection. However, achieving a consistent expression system through the application of Cas9 plasmids in fungal species poses challenges. Hence, a new genome-editing methodology was established. It involved the temporary use of refined CRISPR/Cas9 ribonucleoprotein, pre-added with guide RNA, for specific genetic modification in rice blast pathogen (Foster et al., 2018). This approach, devoid of plasmids, can also be leveraged for the genetic manipulation in various fungi. Consequently, the precise alteration of host genes using gene knock-out or knock-in approaches along with base editing strategies, without causing off-target effects, remains an ongoing imperative (Arazoe, 2021). Thus, continuous enhancement of GET is crucial for rapid and adaptable genome modification of host rice plants and its numerous pathogens.

## 6 Applications of genome editing in creating novel alleles

Genetic mutation represents an evolutionary mechanism responsible for generating allelic variability in plant systems. The inherent mutation frequency in nature is notably low, often failing to induce novel traits in the plants (Kumar et al., 2010). However, rising technological innovations like Next-Generation Sequencing (NGS) enable the creation of comprehensive genomic datasets, which are subsequently analyzed using bioinformatics software to detect novel alleles. Furthermore, confirming the functionality of detected alleles is essential, with CRISPR technologies significantly expediting this confirmation process. For instance, allele surveying for blast resistance to *M. oryzae* in rice led to the discovery of more potent versions of known blast resistance genes (Thakur et al., 2013a; Thakur et al., 2013b; Thakur et al., 2015). The CRISPR-platform can be utilized to create double-strand nicks in the targeted DNA segment through its error-prone base repair mechanism, thereby facilitating the creation of new alleles (Pacher and Puchta, 2017).

### 6.1 Creating novel alleles in rice for advancing biotic tolerance

Numerous studies have aided understanding about how to apply genome editing techniques to generate novel alleles. In rice research, for instance, CRISPR/Cas has been instrumental in creating novel alleles linked to increased yields and resistance towards diseases such as rice leaf blight and rice blast (Zhou et al., 2018; Zhou et al., 2022). Zhou et al. (2018) identified and characterized a rice mutant, *bsr-k1*, which provides a high disease resistance towards both pathogens *Xoo* and *M. oryzae*. The deactivation of the *Bsr-k1* gene results in the accumulation of *OsPAL1-7* mRNA transcripts in the mutant, leading to improved rice yield with enhanced resistance to leaf blight and blast diseases (Zhou et al., 2018). Furthermore, Zhou et al. (2022) introduced mutations in specific genes, namely, *Pi21*, *Bsr-d1*, and *ERF922*, within the rice lines, longke638S and TGMS (male sterile line). The resulting mutated lines displayed increased resistance towards rice blast in contrast to the wild type, demonstrating the potential for multi-gene editing in disease resistance. This genetic manipulation technique facilitates targeted alterations in the DNA sequence, potentially resulting in changes in the traits or characteristics of rice varieties.

Moreover, Macovei et al. (2018) identified novel alleles for resistance to rice tungro disease (RTD) by mutating the *eIF4G* factor gene of IR64 rice variety, susceptible to tungro visus, using CRISPR/Cas9 editing technology. The identified *eIF4G* alleles with mutations close to specific residues conferred resistance to RTD, offering potential targets for breeding resistant varieties. These studies underscore the successful creation of novel alleles in rice through genome editing techniques, highlighting the versatility and effectiveness of CRISPR/Cas in improving rice biotic tolerance traits. Continued research in this domain holds promise for addressing global challenges in food security and sustainable rice production.

Furthermore, the disruption of *OsCPR5.1* in a rice cultivar Kitaake conferred a high level of resistance to rice yellow mottle virus (RYMV) isolate BF1 without any yield penalty (Arra et al., 2023). This study suggests that introducing novel mutations into rice cultivars using CRISPR/Cas technology can be exploited to obtain resistance towards rice yellow mottle virus, potentially facilitating accelerated breeding applications to develop resistance in elite cultivars. Likewise, in rice, an allelic variant of the *SF3B1* splicing factor demonstrated resistance to the herbicide herboxidiene, acts as a splicing inhibitor (Butt et al., 2019). Recently, targeted evolution of *OsACC* and *OsALS* within rice germplasm through a dual Base Editor (BE) discovered new allelic variations that provide herbicide tolerance (Li H. et al., 2020; Kuang et al., 2020). Likewise, a novel mutant wx allele of the rice waxy gene was created using a BE, altering the content of amylose in the mutant plants for the development of soft rice (Xu et al., 2020). Though establishing a platform for selecting desired traits with allelic evolution poses challenges, applying a parallel approach for generating *in vivo* allele in various crop plants presents an intriguing avenue for exploration.

### 6.2 CRISPR-based approaches to rediscover lost crop alleles

In addition, functional genomics approaches based on the CRISPR platform open up the prospect of revisiting neglected under-utilized genetic materials to rediscover alleles that were lost through domestication process. Throughout domestication, several crop species shed certain genes deemed unnecessary for survival but potentially governing key agronomic attributes. A myriad of various alleles controlling phenotypic traits were consolidated in domesticated crops through traditional breeding methods aimed at enhancing the fundamental attributes, such as growth, yield, tolerance to abiotic and biotic stress problems, etc. (Jacob et al., 2018). The non-domesticated wild members of cultivated crops acted as gene pool reservoirs and were utilized for hybridization of desired traits in breeding programs. However, a major drawback of traditional breeding programs is the lengthy time needed and the loss of associated genes due to the genetic hitchhiking effect, decreases heterozygosity (Smith and Haigh, 2008). The advent of CRISPR technology has spurred interest in the domestication of non-domesticated plants through introduction of domestication genes, a process referred to *de novo* domestication (DND), which offers a novel approach to adapting plants for cultivation (Osterberg et al., 2017; Fernie and Yan, 2019). Technological advancements based on CRISPR have demonstrated prospective in the DND of wild relative plants harboring a desired trait without sacrificing genome diversity. For example, Zsogon et al. (2018) achieved successful domestication of wild tomatoes, improving nutritional qualities and yield quantity by employing CRISPR approaches to target various genes including those related to fruit shape, fruit weight, lycopene beta cyclase, and self-pruning. In a parallel manner, the speedy domestication of wild strawberry tomato (*Physalis pruinosa*) was achieved by editing the *CLV1* (receptor kinase) and *SP5G* (associated with flower growth and development) genes using CRISPR/Cas9, leading to enhanced yield in the domesticated plants (Lemmon et al., 2018). Additionally, with the application of CRISPR/Cas9, desirable traits were introduced into wild tomatoes by aiming the *SlWUS*, *SlCLV3*, *SP5G*, and *SP* genes (Li T. et al., 2018). In a recent investigation, the CRISPR approach was utilized to investigate salt resilience in rice and tomato, uncovering *SlHAK20* as a vital factor controlling K+/Na+ homeostasis and contributing to the diminished salt resistance observed in domesticated plants (Wang et al., 2020). This *SlHAK20* represents the initially recognized gene involved in the domestication process linked to decreased tolerance to salt stress, offering valuable molecular insights for enhancing other crops. Furthermore, in rice, Abdullah et al. (2022) discussed the prospective and potential of the DND of wild species to enhance global rice production. They emphasized the advantageous traits found in a wild rice (*Oryza australiensis*), accessible genomic data, along with the capability of genome editing, to uncover and comprehend the roles of novel beneficial alleles. The wild rice domestication through the DND approach holds a lots promise in the crop improvement (Li T. et al., 2018). This process requires the advancement in well-annotated reference genome sequence, efficient transformation method, and the genetic manipulation of various core genes essential for domestication. These genes govern traits like panicle architecture, awn length, shattering attribute, and nutritional quality benefits, aiming to enhance various features.

Applications of genome editing in creating novel alleles have revolutionized research in genetics and crop improvements. The GETs, especially the CRISPR/Cas platform, enable precise modifications of DNA sequences, offering unprecedented control over the genetic makeup of organisms. Genome editing allows scientists to introduce or modify specific useful phenotypes in crops, such as enhanced nutritional value, high tolerance against biotic stresses, and improved yield quality. Creating novel alleles for stress tolerance is crucial in the face of climate change. Genome editing has been used to develop crops with enhanced tolerance to drought, salinity, and other environmental stressors, contributing to more resilient and sustainable agriculture. Genome editing allows researchers to create targeted mutations in specific genes, enabling the study of gene function. By creating novel alleles associated with gene knockout or overexpression, scientists can elucidate the role of genes in various biological processes. Novel alleles generated through genome editing can be used to engineer metabolic pathways in microorganisms for compatible host-pathogen interaction processes, biofuel generation, pharmaceuticals, and other valuable compounds. In conclusion, the applications of genome editing in creating novel alleles extend across diverse fields, promising advancements in agriculture, medicine, and basic research. Ongoing research and technological developments in genome editing techniques continue to broaden the scope of possibilities for creating precise and tailored genetic modifications.

## 7 Challenges and limitations of GET in crop improvement

Genome editing in plants for resistance genes has shown tremendous potential, but it is not without challenges and limitations. Understanding these issues is crucial for addressing them and advancing the application of GETs in plant breeding. Below, we discuss some of the major challenges and limitations of genome editing.

### 7.1 Off-target effects of CRISPR system

One of the most contentious criticisms directed at CRISPR technology revolves around the potential risk of inadvertently mutating non-target genes within the manipulated organism, which could result in unintended biological repercussions within its ecosystem. These random mutations might activate undesirable genes, including those associated with disease susceptibility. Furthermore, gene editing procedures could induce translocations of chromosomal segments, leading to genome instability. To mitigate the risks of off-target effects associated with Cas9, numerous strategies have been devised, primarily focusing on optimizing the design of sgRNA. Studies have demonstrated that the utilization of truncated sgRNA can diminish undesired mutagenesis at certain off-target sites while maintaining high efficiency in on-target genome editing (Ding et al., 2016). Additionally, Muller et al. (2016) introduced a *Streptococcus thermophilus* Cas9 variant featuring a longer PAM sequence, resulting in reduced off-target activity. Another promising avenue for precision genome engineering involves the nmeCas9 system, which offers a safer alternative, albeit with slightly lower efficiency compared to spCas9 (Lee et al., 2016). Paired Cas9 nickases represent another approach, generating two single-strand nicks occurring on DNA strands, thus exhibiting high specificity and efficacy without off-target activity (Cho et al., 2014). Regulating Cas9 off-target activity is also achievable by selecting appropriate sgRNA sequences and optimizing experimental conditions (Doench et al., 2016).

### 7.2 CRISPR-component delivery methods

The transfer of CRISPR/Cas cargoes presents a significant difficult task in the application of plant GET, particularly in monocotyledons, where gene gun and agrobacterium-directed delivery effectiveness heavily depend on the recipient genotype. Notably, certain elite rice cultivars pose difficulties in transformation due to inherent limitations in their culture and regeneration capabilities (Zeng et al., 2020). Furthermore, the inevitable incorporation of T-DNA plasmid fragment followed by plant regrowth and development processes is often practically intricate and labor-intensive. Hence, the development of delivery methods that circumvent the need for tissue culture is desirable, extending their applicability in different plant systems. Nanotechnology derived materials like carbon nanotubes (CNTs) and ultrafine particles or nanoparticles (NPs) offer a promising avenue for the CRISPR/Cas system, facilitating the diffusion of desired DNA into plants passing cell wall barriers without any external assistance (Tao et al., 2021). In 2017, a novel approach termed pollen magnetofection (PMF) utilized magnetic NPs as carriers of genetic material to introduce foreign genes into the pollen of multiple model crops, resulting in approximately 1% generation of transgenic plants upon pollination with magnetofected pollen (Zhao et al., 2017). However, concerns regarding the reproducibility of pollen magnetofection have been raised by some researchers (Wei et al., 2021). If the contents of CRISPR/Cas could be effectively delivered to reproductive cells and reliably expressed through the PMF method, it could offer a streamlined method for creating heritable genetic modifications in transgenic seeds, eliminating the necessity of tissue culturing step (Molla et al., 2016). Additionally, owing to the integration and disease free properties of nano genetic transportation approaches, gene-edited crops mediated by nanomaterials may potentially avoid classification as genetically modified organisms (GMOs; Xu et al., 2017).

### 7.3 Ethical considerations in genome editing based crop improvement

GETs, particularly CRISPR/Cas, have revolutionized crop improvement, offering powerful tools for targeted genetic modifications. However, the ethical considerations surrounding the application of genome editing in agriculture are paramount. Balancing the potential benefits with ethical concerns is crucial for responsible and sustainable use. Some key ethical considerations, along with relevant references, include environmental impact, social justice and access, transparency and communication, consumer acceptance and labelling, unintended consequences and long-term effects, dual-use and bioweapon concerns, and respect for biodiversity and traditional farming practices.

The unintended environmental consequences of genome-edited crops, such as gene flow to wild relatives or the potential disruption of ecosystems, raise ethical concerns. Evaluating and minimizing the environmental impact of genome-edited crops through rigorous risk assessments and containment measures is essential (Pauwels et al., 2014). Ensuring equitable access to GETs and their benefits, especially for smallholder farmers in developing countries, raises concerns about social justice. Addressing issues of access and distribution to avoid creating disparities and ensuring that benefits are shared globally is crucial for ethical genome editing applications in agriculture (Hilbeck et al., 2020). Maintaining transparency in research, development, and deployment of genome-edited crops, along with effective communication with stakeholders, is essential for ethical practice. Establishing open communication channels and involving the public in decision-making processes can build trust and address concerns related to transparency (Kuzma et al., 2016).

Ethical concerns arise regarding consumer acceptance and the labeling of genome-edited products, as some consumers may have reservations about genetically modified organisms (GMOs). Implementing clear labeling practices, coupled with effective public engagement and education, can empower consumers to make informed choices and address ethical concerns (Kjeldaas et al., 2023). The potential for unintended consequences and long-term effects of genome editing, including off-target mutations and ecological impacts, necessitates careful ethical consideration (Waltz, 2016). The dual-use nature of GET raises ethical concerns about its potential misuse for bioweapon development or harmful purposes. Implementing responsible governance frameworks, international cooperation, and strict regulations can help prevent the misuse of GETs (Ienca and Vayena, 2018). The potential impact of genome-edited crops on biodiversity and traditional farming practices requires careful ethical consideration to avoid unintended consequences. Respecting and preserving biodiversity, as well as engaging with local communities to understand their perspectives and needs, is crucial for ethically sound genome editing practices (Ishii and Beriain, 2019). Ethical concerns regarding GETs utilization in crop development demand a multidisciplinary approach, involving scientists, policymakers, ethicists, and the public. Engaging open dialogue, transparent practices, and adherence to ethical principles are crucial for responsibly navigating the complex landscape of genome editing in agriculture.

### 7.4 Regulatory frameworks for GET

Genome editing with designed nucleases emerges as an extremely precise and effective method for enhancing crops, offering the possibility to swiftly create new beneficial traits. These techniques stimulate precise double-strand nicks in the genome, initiating DNA repair pathways that can result in base insertions, deletions via NHEJ, or precise gene swapping and transgene inclusions by HDR systems. The phenotypic variations produced by these techniques often mimic those arising through natural processes or conventional mutagenesis, blurring the lines with exiting definitions of genetically modified or engineered organisms under prevailing regulatory frameworks. The ambiguity surrounding the regulatory reputation of genome editing approaches poses a significant hurdle to their widespread adoption for economically valuable crop qualities. Regulatory emphasis on the methodology employed, instead of the characteristics of the resulting phenotype, has led to confusion among regulators, developers, and the public, fostering uncertainty regarding the utilization of genome manipulation methods in crop development programs. The regulatory landscape concerning genome-edited crops differs globally, contributing to uncertainties in their classification and acceptance (Wolt et al., 2016). Establishing clear and globally harmonized regulations for genome-edited crops, while addressing safety concerns and promoting public acceptance, is imperative for their integration into agricultural practices.

Several challenges are associated with GET, including genetic mosaicism, complex trait regulation, and epigenetic changes. Genetic mosaicism, characterized by the coexistence of edited and unedited cells within a tissue, can complicate the phenotypic analysis of edited plants. Careful screening and selection of plants with desired edits, coupled with advancements in editing efficiency, can help mitigate the impact of genetic mosaicism (Fauser et al., 2014). Resistance to certain plant diseases or pests often involves complex genetic pathways, posing challenges in achieving desired traits through single-gene modification. Employing multiplex genome editing strategies to target multiple genes involved in resistance pathways can enhance the effectiveness of genome editing for complex traits (Staskawicz et al., 1995). Genome editing may induce unintended epigenetic changes, potentially affecting gene expression and overall phenotype. Further research into understanding and minimizing epigenetic alterations, alongside improved design and delivery of editing tools, is necessary (Niederhuth et al., 2016). Addressing these challenges and limitations will require ongoing research, technological advancements, and collaborative efforts among scientists, policymakers, and the public to completely harness the potential of genome editing for developing resistant crops and ensuring global food security.

## 8 Future prospects of GETs in the plant disease resistance

The future of GETs for enhancing plant disease resistance holds great promise, with continuous advancements aimed at addressing global challenges in agriculture. Emerging technologies are expanding the precision, efficiency, and scope of genetic modifications for enhancing plant resistance against diseases. In genome editing, prime editing and base editing technologies offer enhanced precision by enabling the direct alteration of one DNA nucleotide to another ones without introducing double-strand nicks. These technologies reduce the likelihood of unintended mutations and provide more accurate control over the modification of specific nucleotides associated with disease resistance genes (Komor et al., 2016; Anzalone et al., 2020). Likewise, epigenome editing enables for the precise alternation of epigenetic marks, such as DNA base methylation and histone changes, influencing gene expression devoid of altering the underlying DNA base sequence. Targeting epigenetic regulation provides a nuanced approach to modulating gene expression for enhanced disease resistance, offering potential benefits in crop protection (Kearns et al., 2014). RNA editing technologies, such as CRISPR/Cas13, enable the targeted modification of RNA sequences, offering a versatile method for controlling gene expression post-transcriptionally. RNA editing can be applied to fine-tune the expression of disease resistance genes, providing an additional layer of control and adaptability (Abudayyeh et al., 2017). Multiplex editing strategies and synthetic biology approaches enable the simultaneous modification of multiple genes or the engineering of custom genetic circuits to enhance disease resistance. This allows for a comprehensive and tailored approach to fortifying plants against various pathogens and environmental stresses (Lowder et al., 2015). Innovations in delivery methodologies, like nanoparticles, nanotubes, and viral vectors, aim to advance the efficiency of introducing genome editing components into plant cells. Enhanced delivery methods contribute to overcoming challenges related to transformation efficiency, especially in crops with traditionally low transformation rates (Liu et al., 2019). Integrating CRISPR screening combined with genome-wide association analyses helps us in the identification of novel genes and regulatory elements associated with disease resistance. This approach facilitates a systems-level understanding of plant-pathogen interactions and provides insights for engineering durable resistance in crops (Zhang Y. et al., 2019; Wei et al., 2020). Machine learning and predictive modeling techniques provide the potential to accelerate the discovery of candidate genes as well as design optimal genome edits for improved disease resistance. Integration of computational tools with GETs enhances the efficiency and precision of engineering plants with robust resistance towards specific pathogens (Singh et al., 2021). However, a few questions should be addressed in the coming times for the effective utilization of GETs in crop improvement programs. For instance, is there room for enhancing transformation systems not reliant on tissue culture to effectively transfer CRISPR components into plant cells, especially for plants not considered as a model system? Furthermore, is it possible to design a system that creates transversion-type modification? In summary, the future prospects of GETs for plant disease resistance involve a convergence of innovative tools, advanced delivery systems, and interdisciplinary collaborations. These emerging technologies hold the potential to revolutionize crop protection strategies, contributing to sustainable agriculture and global food security. Continued research, ethical considerations, and stakeholder engagement will be essential for harnessing the full potential of these technologies.

## 9 Conclusion

In conclusion, the exploration of harnessing the full potential of genome editing in rice resistance genes underscores the transformative possibilities that genome editing brings to the realm of agriculture. With advancements in technology, particularly in CRISPR/Cas, we stand at the precipice of a new era where we can precisely manipulate and create novel allele of rice resistance genes to enhance crop resilience and yield. The potential benefits are immense, from mitigating the impact of pests and diseases to ensuring global food security. However, ethical considerations, regulatory frameworks, and ongoing research are crucial aspects that need careful attention to navigate this promising but complex landscape. As we continue to unlock the mysteries encoded in the rice genome, responsible and sustainable deployment of genome editing techniques will be essential to harness its full potential for the betterment of agriculture and society as a whole.
